# Nutrition Knowledge, Attitudes, and Practices (KAPs) among Jordanian Elderly—A Cross-Sectional Study

**DOI:** 10.3390/nu15092220

**Published:** 2023-05-08

**Authors:** Fadwa Hammouh, Mai Abdullah, Ala’a Al-Bakheit, Narmeen Jamal Al-Awwad, Ibrahim Dabbour, Ayoub Al-Jawaldeh

**Affiliations:** 1 Nutrition and Dietetics Department, Faculty of Health Sciences, American University of Madaba, Madaba 11821, Jordan; 2 Department of Nutrition and Food Technology, The University of Jordan, Amman 11942, Jordan; 3 Department of Nutrition and Food Processing, Faculty of Agricultural Technology, Al-Balqa Applied University, Salt 19117, Jordan; 4 Department of Clinical Nutrition and Dietetics, Faculty of Applied Medical Sciences, The Hashemite University, Zarqa 13133, Jordan; 5 Department of Nutrition and Food Processing, Faculty of Agriculture, Mu’tah University, Karak 61710, Jordan; 6 Regional Office for the Eastern Mediterranean, World Health Organization, Cairo 7608, Egypt

**Keywords:** elderly, nutrition, knowledge, attitude, behavior, education

## Abstract

Nutrition knowledge, attitudes, and good practices are essential for preventing malnutrition, ensuring good health, and maintaining life quality. However, to the best of our knowledge, no studies have been published on the nutritional knowledge, attitudes, and practices (KAPs) of Jordanian older people. For this reason, our study aimed to assess the KAPs in the Jordanian elderly. A cross-sectional survey was conducted among 1200 people aged 60 and over. The results revealed that 52.8% of participants had poor knowledge, 52.7% had negative attitude scores, and 72.6% had poor practices. Significant differences were found between the three regions in the KAP prevalence (*p* < 0.001). The northern region had a higher prevalence of nutritionally poor knowledge (65.6%) compared to 52.5% and 40.4% for the central and southern regions, respectively. Participants from the central region had a higher prevalence of a positive attitude (55.4%), whereas the northern and the southern participants had a higher prevalence of a negative attitude (65.6% and 54.4%, respectively). All regions reported poor practices, yet, significantly, the northern regions had the highest prevalence of poor practices. Participants with a low educational level reported a significantly higher prevalence of poor knowledge, negative attitudes, and poor practices compared to participants with a high educational level. The results obtained underline the importance of taking into account the lack of nutrition-related KAPs among the elderly in Jordan. It is crucial to raise awareness on this issue and to implement the national nutrition strategy, with particular attention paid to the elderly. Concrete measures must be taken to ensure that the nutritional needs of older people are met and to improve their quality of life.

## 1. Introduction

The World Health Organization (WHO) and the United Nations have defined the aging population as those with a chronological age of 60 years and over [1,2,3,4]. The age cut-off definition of old age is the basis of establishing multiple political decisions, such as determining the retirement age [2]. In Jordan, older adults (elderly) are 60 years and above [5]. The Jordanian Department of Statistics [6] documented in 2021 the elderly population as being 588,075 compared to 405,101 reported in 2016, with a 45% increase over the 5 years [5]. Among the elderly population, 288,615 (49.1%) are women and 299,460 (50.9%) are men [6]. Worldwide, it has been stated that by 2050, the elderly population will have kept growing [7] and is expected to reach two billion, meaning that more than 20% of the world’s population will be 60 years and over [8].

The results of the Jordanian National STEPs survey [9] on non-communicable disease (NCD), cardiovascular disease (CVD), cancer, diabetes mellitus (DM), and chronic respiratory disease (CRD) risk factors showed that NCDs are the main cause of morbidity and mortality in this country, constituting 78% of all deaths [10]. In particular, according to 2021 WHO data, CVD is the leading cause of death in Jordan, accounting for approximately 45% of all causes of death in the country [11]. A recent study concluded that CVD risk factors and their clusters are highly prevalent among older male participants [12], whereas dyslipidemia is the most prevalent modifiable risk factor clustering with other CVD risk factors [13]. Dietary issues and lifestyle are therefore vital in preventing CVD progression.

Malnutrition is a crucial health concern in the elderly [14,15,16]. It can indicate either undernutrition, which correlates with nutrient deficiencies, or overnutrition, which results from consuming more energy than recommended, leading to obesity [15,16,17]. Obesity, in particular, has been linked to several chronic diseases, including DM, metabolic syndrome, CVDs, and cancer. It has been shown that the percentage of the elderly with chronic diseases is very high [14,15].

Nutrition knowledge, attitudes, and good practices (KAPs) are essential for preventing malnutrition and ensuring good health [18]. Good nutrition knowledge is positively correlated with good nutritional status. A desire for healthy and balanced consumption may not entirely translate into behavior modification. The continuous consumption of unhealthy foods leads to serious health problems. Achieving desired behavior changes in health and nutrition depends on acquiring sufficient knowledge, attitudes, good practices, and self-efficacy [19]. Behaviors can be assessed through KAPs that can be updated to evaluate the effectiveness of intervention programs [19]. It has been reported that the lack of knowledge and awareness regarding hypertension contributes to an increase in its prevalence, which may increase the risk of complications related to this pathology in the elderly [20]. In addition, it should be noted that older people have been under-represented in research and nutrition awareness campaigns conducted in Jordan. In this context, the study aims to develop a comprehensive and structured methodology to evaluate the nutrition KAPs among the elderly in Jordan. We suggest that the results be used to assess the effectiveness of prevention and intervention programs, and further assist decision-makers to develop policies to address issues associated with obesity, hypertension, micronutrient deficiencies, and NCDs in the elderly, with a focus on reducing excessive calorie, sugar, salt, and saturated fat consumption which should be incorporated into national public health strategies.

## 2. Materials and Methods

### 2.1. Ethical Approval

The study protocol was submitted to the Ethics Committee of the Institutional Review Board (IRB) of the Faculty of Health Sciences, American University of Madaba (approval number: H21004) and was approved prior to initiation. The study was conducted in strict accordance with the Declaration of Helsinki and written consent was obtained from all of the participants. To preserve their anonymity and confidentiality, the data collected were processed anonymously and no information-identifying participant data were collected.

### 2.2. Study Design

A cross-sectional study was conducted in three discrete regions (north, central, and south) covering all Jordanian governorates to determine the KAPs related to nutrition in the elderly for the period between December 2021 and March 2022. The study population included people aged 60 years and over residing in the study area. The number of subjects was equal to and not less than 1200 for significance. According to the Department of Statistics, 588,075 of the Jordanian population are ≥60 years old [6]. The inclusion criteria were (1) age ≥ 60 years, (2) Jordanian, (3) awareness and ability to understand and respond correctly to the questions, and (4) willingness to participate in the study and sign the informed consent form.

### 2.3. Questionnaires

A structured questionnaire was developed from a literature review and translated to Arabic [20] (see Supplementary Files S1 and S2). Prior to the study, the content of the questionnaire was assessed by a panel of academic nutrition experts to ensure its validity. To assess the reliability of the questionnaire, a pilot study was conducted among 42 Jordanian elderly people, who completed it twice with an interval of 3 weeks. Questionnaire reliability was then assessed based on the guidelines published by FAO in 2014 [21]. The KAP questionnaire reliability was assessed using Cronbach’s alpha test. Cronbach’s alpha values were 0.701, 0.943, and 0.805 for knowledge (K), attitude (A), and practice (P) scores, respectively. The questionnaire was divided into four distinct parts. The first part concerned the general characteristics of the study sample, in which data were collected on socio-demographic variables, body mass index (BMI) categories, smoking, sports habits, consumption frequency of the three main meals, and the health history of the participants.

Parts two, three, and four of the questionnaire assessed participants’ knowledge, attitude, and practice, respectively. Knowledge was assessed via nine closed-ended questions (yes/no), while attitude was assessed via nineteen multiple-choice questions (strongly agree, agree, fair, disagree, strongly disagree). The attitude questions were designed to measure the participants’ emotional, motivational, perceptual, and cognitive beliefs related to their diet that may have influenced their behavior or practices (attitudes). Finally, the practice was evaluated via nine closed questions (yes/no) relating to the observable actions of participants that could have had an impact on their nutrition, such as eating or feeding.

### 2.4. Scoring of Data

The nutritional knowledge score was assessed using the attribution method. Each question was scored on the basis of a “yes” or “no” response, and correct responses were awarded one point, while incorrect responses received a score of zero. The entire section was worth a total of 9 points, and the raw scores were then converted to a percentage. Levels of nutritional knowledge and practices were classified into 3 categories: poor (0–50%), moderate (51–74%), and good (˃75%).

The attitude scale consisted of 19 questions. Responses were rated using a five-point Likert scale ranging from (1) strongly disagree to (5) strongly agree. The total scores ranged from 19 to 95. The lower scores showed a negative attitude toward nutrition [22]. Overall attitude scores were classified into 3 classes:

- Negative: <71.70%;
- Neutral: 71.70–84.92%;
- Positive: >84.92%.

To obtain the scores for K, A, and P, the scores for each question in the corresponding section were added together, with maximum possible scores of 6, 3, and 10, respectively. The total score for the questionnaire was 19, calculated by adding the K, A, and P scores. A higher score indicated better KAPs in nutrition of the patient [23,24].

### 2.5. Statistical Analysis

Data obtained from 1200 participants were systematically consolidated and transformed into a double-checked master-coding sheet before being analyzed. Statistical analyses were performed using IBM® SPSS® Statistics software (version 24.0 for Windows, Armonk, NY, USA, Released 2016) [25]. For continuous variables, the mean and standard error of the mean (SEM) were calculated using a descriptive test, while categorical variables were described using frequencies and percentages via the chi-square test. Differences between male and female participants in terms of knowledge, attitudes, and practices were detected using an independent samples T-test, while differences between regions were examined using a one-way ANOVA test. A value of *p* < 0.05 was considered to be statistically significant.

## 3. Results

In this study, we gathered a total of 1200 participants, equally distributed in the different regions of Jordan (33.2% in the center, 33.4% in the north, and 33.4% in the south). The socio-demographic data, BMI, sports habits, smoking, frequency of the three main meals, and the medical history of the participants are presented in Table 1. Almost half of the sample participants were women (50.2%), and the majority of whom (70.8%) lived in cities. Most participants (92.2%) were between 60 and 79 years old, with only 7.8% over the age of 79. Among the entire sample, 73.3% of the participants were married and 87.6% lived with their spouse or family. In addition, most of the participants were poorly educated (42.3%), unemployed (80.4%), and had an income of less than JD 500 (61.0%), depending mainly on a retirement salary to support themselves (57.6%). In contrast, the majority of the participants had a high BMI, with 38.7% being overweight and 34.6% being obese, and 76.0% of them were smokers. Furthermore, a large majority of the participants (88.5%) considered that the elderly were able and should exercise regularly. Regarding eating habits, 87.9% of the participants had breakfast, 92.4% had lunch, and 65.7% had dinner.

When it came to oral and vision health, 51.0% and 61.7% of participants reported problems, respectively, with 40.0% reporting problems with long vision. Finally, the majority of participants (74.6%) suffered from chronic illnesses.

The knowledge, attitude, and practice scores among the selected participants and their prevalence are illustrated in Table 2. Regarding knowledge, more than half of the participants (52.8%) declared having poor knowledge. For attitude, nearly half of the participants (52.7%) obtained negative scores. Finally, with regard to practice, two thirds of the participants (72.6%) had poor practice.

In Table 3, the prevalence of knowledge, attitudes, and practices according to gender is illustrated. There were no significant differences between men and women in scores on knowledge, attitude, and practices. However, both genders reported poorer knowledge, negative attitudes, and poorer practice prevalence.

Table 4 shows participants’ knowledge, attitude, and practice prevalence by region. Significant differences existed between the three regions in terms of knowledge, attitude, and practice prevalence (*p* < 0.001). Indeed, the prevalence of nutritionally poor knowledge was highest in the northern region (65.6%), followed by the central (52.5%) and southern (40.4%) regions. However, it should be noted that the southern region had a higher prevalence of good knowledge (59.6%) compared to the other regions. Concerning attitude, participants from the central region had a higher prevalence of positive attitudes (55.4%), while those from the northern and southern regions had a higher prevalence of negative attitudes (65.6% and 54.4%, respectively). For practices, all regions reported high rates of poor practices. However, the northern region had the highest prevalence of poor practices (80.5%), followed by the central (72.8%) and southern (64.4%) regions. This difference was statistically significant (*p* < 0.001).

Regarding the results of the prevalence of nutritional KAPs in different regions, by sex, there were no significant differences among the central regions between men and women with regard to their nutritional KAPs, with a high prevalence of poor knowledge, positive attitude, and poor practice for both genders. In the northern region, no significant differences were observed between men and women regarding nutritional KAPs, with a high prevalence of poor knowledge, negative attitudes, and poor practices in both genders. For the southern region, there was no significant difference between men and women regarding nutritional knowledge; both reported a high prevalence of good knowledge. However, it should be noted that the results of the study revealed significant differences between the attitudes and practices of men and women. Indeed, women reported a significantly higher prevalence of negative attitudes (58.2%) than men, who in turn reported a higher prevalence of positive attitudes (52.6%, *p* < 0.001). For practices, women reported a significantly higher prevalence of poor practices (68.4%) than men (58.8%).

On the other hand, Table 5 presents the participants’ knowledge, attitude, and practice prevalence based on their educational level. Participants with a low educational level (no education, high school, and college diploma) reported poor knowledge significantly more often (55.0%, 56.3%, and 53.5%, respectively) than participants with a high educational level (bachelor’s degree and higher), who declared significantly good knowledge (53.6% and 75.0%, respectively). Similar results were seen in attitude prevalence. In fact, participants with a low educational level (no education, high school, and college diploma) reported a significantly higher negative attitude (57.9%, 53.7%, and 53.0%, respectively) compared to participants with a high educational level (bachelor’s degree and higher) who reported having a significantly higher positive attitude (59.1% and 70.3%, respectively). Regarding practices, participants with a low educational level (no education, high school, and college diploma) reported significantly poorer practices (76.5%, 69.7%, and 74.8%, respectively) compared to participants with a high educational level (bachelor’s degree (69.4%) and higher (51.3%)).

## 4. Discussion

KAP surveys are the most commonly used study tools to investigate dietary patterns associated with health risks. They are descriptive of a particular population, which involves gathering information about what is known, believed, and done concerning a specific topic. The objective of our study was to design a comprehensive and well-structured methodology to assess the nutritional KAPs of the elderly residing in Jordan. In fact, we evaluated the knowledge of the elderly population to understand the extent to which community knowledge relates to nutritional concepts and nutritional status. At the same time, attitude indicates the thoughts as well as feelings of individuals toward nutritional concepts and any predetermined ideas that they may have [26], while practice indicates how they determine their knowledge and attitude through their actions.

Attitudes toward nutritional concepts were evaluated by asking positively or negatively worded questions, as were practices, which were assessed using questions labeled as representing good or poor practice. The KAPs of the population are proportional to the prevention of any disease and reflect the societal importance placed on health-related issues. Good nutritional knowledge and a positive attitude affect dietary practices, leading to good nutritional status and disease prevention. The results presented in this study point to important health issues among the older adults participating in the study; nearly half of the participants reported having poor knowledge and negative attitude scores, while two thirds (72.6%) had poor practice, as shown in Table 2. These results are alarming as they indicate a high prevalence of modifiable risk factors for CVD among participants. Our results are in agreement with those obtained in the study by Alsaud and colleagues, which showed a high prevalence of CVD risk factors among Jordanians, in particular regarding dyslipidemia [13]. According to these results, chronic diseases associated with the elderly appear to be prevalent. This was consistent with our data that showed that nearly two thirds of participants suffered from chronic conditions (Table 1). Moreover, the high rates of overweight (38.7%) and obese (34.6%) individuals compared to those of normal weight (25.6%) and underweight (1.1%) individuals in our study, as presented in Table 1, are of concern. Obesity and being overweight are forms of malnutrition that often reflect poor nutritional knowledge and, thus, poor practices among the elderly.

Moreover, the results of our study indicate that participants’ knowledge in the southern region was good and was proportionally related to their health attitudes and practices. Indeed, several studies have shown that health knowledge, attitudes, and practices can vary from one region to another [27,28,29,30]. This suggests that it may be important to understand regional differences in health/nutritional KAPs in order to design effective public health programs that can meet the specific needs of each region.

Furthermore, in the northern region, knowledge was rated poor (Table 4). This difference can be explained by socio-economic factors such as poverty and cultural beliefs. Analyzing the characteristics of the participants in our study, we found that 80.4% of them were not working and had an income of less than JD 500 (61.0%), with retirement salary being the primary source of income (57.6%). Additionally, a majority of the participants were obese (38%), and a large proportion of them had no formal education (42.3%). These findings highlight the importance of considering socio-economic and cultural contexts in efforts to improve health literacy in the northern region. Poverty can be a factor that limits access to education and educational resources, which could explain the lack of knowledge in this region. Additionally, cultural beliefs can also play an important role. In some cultures, it is considered inappropriate for women to pursue formal education, which could limit their access to information and knowledge. However, it should be noted that these results may not be generalizable to the whole country or even to other countries. It is also important to note that the correlation between poverty and the level of knowledge is not always clearly defined and that there are other factors to consider, such as the quality of education and learning opportunities available. Indeed, several investigations have recorded the impact of socio-economic and cultural factors between regions on the health status of older adult men and women.

In contrast, people with low education or limited knowledge of nutrition may be influenced by cultural beliefs that are not necessarily backed by science to achieve a healthy weight [31]. In addition, some nutritional practices promoted by inexperienced dietitians and healthcare professionals, such as selecting expensive and out-of-season foods, can make adopting healthy eating behavior seem costly [32]. This perception can lead community members to look for alternative ways to reach their healthy weight goal, often based on unscientific practices and associated with many health issues.

Our findings on the effect of knowledge on attitudes and practices are in agreement with those of Hag and colleagues [33]. Poor attitude and practice scores in the elderly population were also observed in the study conducted by Kumar et al. [3].

Although there were no significant differences between men and women regarding knowledge, attitudes, and practices (Table 3), the mean KAP scores were significantly associated with gender in different regions. Our study reveals a significantly more negative attitude score of women than men in the southern region, as well as a high and significant score for poor practices for both sexes in this region. Although this trend was also found in the north and center, the scores were not significant there. These results suggest that there are no significant differences between the knowledge, attitudes, and practices of men and women in the entire sample studied. However, when the results were analyzed by region, significant differences appeared. These findings should be tailored to address the specific challenges faced by different regions and should take into account gender-specific factors. This approach can help to achieve better health outcomes and reduce the prevalence of malnutrition. Interestingly, no study has so far examined the relationship between geographic location and mean KAP scores. Our study is therefore the first to report an association between average KAP scores, gender, and regions.

On the other hand, our findings highlighted a correlation between the average KAP scores and the educational level of participants (Table 5). Indeed, participants with low educational levels reported significantly poorer knowledge, negative attitudes, and poor practices. These results are in agreement with previous research carried out by Cheung et al. [34] and Wu et al. [35], who also found that the level of education was a determinant of KAP scores. In addition, the study by Haron et al. [20] revealed a significant correlation (*p* < 0.05) between a higher educational level and good knowledge scores. It is well established that nutrition knowledge is closely related to education and that adequate knowledge can be achieved as the educational level increases, leading to a positive attitude and, consequently, good dietary practices [33]. Other recent studies carried out in Jordan on different age groups have reinforced these findings by suggesting that structured educational interventions can be an effective way to improve knowledge, attitudes, and practices in the prevention of various health problems [36,37].

Indeed, Elsahoryi et al. [36] evaluated the impact of an educational intervention with the parents of children (2 to 15 years old) with celiac disease. Therefore, improving parents’ level of knowledge about this disease significantly increased their children’s adherence to the gluten-free diet, which had a positive impact on their health status. Similarly, Abu-Baker et al. [37] found that educational interventions had beneficial effects in adolescents with iron deficiency anemia.

Since our study showed that the education level was low, especially among residents of the central and northern regions, it is essential to put in place policies aimed at providing in-depth nutritional knowledge to the general population. Improving nutritional knowledge can contribute to a positive attitude and good dietary practices, which can lead to optimal nutritional status.

## 5. Conclusions and Recommendations

The increase in the elderly population in Jordan highlights the importance of nutrition in improving their quality of life. This study assessed the nutritional knowledge, attitudes, and practices of Jordanian older adults to determine the dietary patterns associated with health risks. The results showed a lack of nutritional knowledge, negative attitudes, and poor practices among the elderly in all regions of the country, although regional differences were observed. However, a positive correlation was established between the level of education and healthy eating practices. It is recommended that these results be used to develop educational programs and national action plans to improve the nutrition of older people in Jordan. Providing in-depth knowledge could lead to improved healthy eating attitudes and practices. Policy makers and health professionals will be able to use these results to target nutrition interventions for the region’s most in need. Furthermore, it is important to continue research to understand the underlying causes and develop effective interventions to improve the nutrition of older people in Jordan.

## Figures and Tables

**Table 1:** Socio-demographic data, BMI, sports habits, smoking practice, dietary habits, and medical history of participants (*n* = 1200).

Variables	*n* (%)	Variables	*n* (%)
Socio-Demographic		Body Mass Index (BMI) Category	
Place of Residence		Underweight	12 (1.1)
City	843 (70.8)	Normal Weight	281 (25.6)
Village	348 (29.2)	Overweight	424 (38.7)
Gender		Obesity	379 (34.6)
Female	600 (50.2)	Smoking	
Male	595 (49.8)	No	912 (76.0)
Age		Yes	288 (24.0)
60–69 yrs.	783 (66.0)	Sports Habits	
70–79 yrs.	310 (26.2)	No	129 (11.5)
≥80 yrs.	92 (7.8)	Yes	996 (88.5)
Marital Status		Breakfast	
Single	59 (5.0)	No	145 (12.1)
Married	870 (73.3)	Yes	1052 (87.9)
Divorced	26 (2.2)	Lunch	
Widowed	232 (19.5)	No	91 (7.6)
Living		Yes	1105 (92.4)
Alone	147 (12.4)	Dinner	
Spouse/Family	1038 (87.6)	No	410 (34.3)
Education Level		Yes	786 (65.7)
Not educated	502 (42.3)	Medical History	
High school	314 (26.4)	Tooth Problems	
College diploma	160 (13.5)	No	587 (49.0)
Bachelor	172 (14.5)	Yes	612 (51.0)
Higher than that	40 (3.4)	Vision Problem	
Work		No	460 (38.3)
No	961 (80.4)	Yes	740 (61.7)
Yes	235 (19.6)	Type of Vision Problem	
Monthly Income Rate		Long vision	274 (40.7)
Less than JD 500	721 (61.0)	Short vision	248 (36.8)
JD 500–1000	399 (33.8)	Cataract	152 (22.6)
More than JD 1000	62 (5.2)	Chronic Diseases	
Income Source		No	304 (25.4)
Work	231 (19.7)	Yes	893 (74.6)
Children	213 (18.1)		
Retirement salary	677 (57.6)		
National aid	54 (4.6)		

**Table 2:** General knowledge, attitude, and practice scores.

Variables	Evaluations and Judgments	*n* (%)
Knowledge	Poor knowledge	620 (52.8)
	Good knowledge	554 (47.2)
Attitude	Negative attitude	596 (52.7)
	Positive attitude	534 (47.3)
Practices	Poor practices	859 (72.6)
	Good practices	325 (27.4)

**Table 3:** Gender-based knowledge, attitude, and practice scores.

Variables	*n* (%)		*p*-Value *
	Female	Male	
Knowledge			
Poor knowledge	313 (53.7)	305 (52.0)	0.574
Good knowledge	270 (46.3)	281 (48.0)	
Attitude			
Negative attitude	312 (54.4)	282 (51.1)	0.272
Positive attitude	262 (45.6)	270 (48.9)	
Practices			
Poor practices	432 (72.7)	424 (72.5)	0.924
Good practices	162 (27.3)	161 (27.5)	

* *p*-value < 0.05 considered to be statistically significant.

**Table 4:** Region-based knowledge, attitude, and practice scores.

Variables	*n* (%)			*p*-Value *
	Central Region	Northern Region	Southern Region	
Knowledge				
Poor knowledge	200 (52.5)	258 (65.6)	160 (40.4)	<0.001
Good knowledge	181 (47.5)	135 (34.4)	236 (59.6)	
Attitude				
Negative attitude	165 (44.6)	226 (59.3)	204 (54.4)	<0.001
Positive attitude	205 (55.4)	155 (40.7)	171 (45.6)	
Practices				
Poor practices	283 (72.8)	318 (80.5)	255 (64.4)	<0.001
Good practices	106 (27.2)	77 (19.5)	141 (35.6)	

* *p*-value < 0.05 considered to be statistically significant.

**Table 5:** Knowledge, attitude, and practice prevalence based on education levels, *n* (%).

Variables	Education					*p*-Value *
	Not Educated	High School	College Diploma	Bachelor’s Degree	Higher	
Knowledge						
Poor knowledge	271 (55.0)	174 (56.3)	83 (53.5)	77 (46.4)	10 (25.0)	0.001
Good knowledge	222 (45.0)	135 (43.7)	72 (46.5)	89 (53.6)	30 (75.0)	
Attitude						
Negative attitude	274 (57.9)	158 (53.7)	80 (53.0)	67 (40.9)	11 (29.7)	<0.001
Positive attitude	199 (42.1)	136 (46.3)	71 (47.0)	97 (59.1)	26 (70.3)	
Practices						
Poor practices	380 (76.5)	214 (69.7)	119 (74.8)	118 (69.4)	20 (51.3)	0.004
Good practices	117 (23.5)	93 (30.3)	40 (25.2)	52 (30.6)	19 (48.7)	

* *p*-value < 0.05 considered to be statistically significant.

## Data Availability

Not applicable.

## Supplementary Materials

The following supporting information can be downloaded at https://www.mdpi.com/article/10.3390/nu15092220/s1, Supplementary Files S1 &S2Questionnaire in English &Questionnaire in Arabic.
