# Supplementary material for: Nutrition Knowledge, Attitudes, and Practices (KAPs) among Jordanian Elderly—A Cross-Sectional Study
Source: Nutrients. 2023 May 8;15(9):2220. doi: 10.3390/nu15092220 (PMC10181286; doi:10.3390/nu15092220)
Supplement: Supplementary file 1 [file nutrients-15-02220-s001.zip › Supplementary File S1.pdf]

<u>Supplementary File- S1</u>
<u>American University of Madaba</u>
<u>Faculty of Health Sciences/ Nutrition and Dietetics Department</u>
Consent form for participation
Nutrition Knowledge, Attitude, and Practice among Jordanian Elderly- A Cross -Sectional Study

IRB Approval No.: ----------------

Respected Participants:

Participation in this study is completely voluntary. If you decide not to participate there will not be any negative consequences.

The researcher will maintain the confidentiality of the research records or data, and all data will be anonymous without any indication for the personal identity.

By submitting this form you are indicating that you have read the description of the study, you are 60 years old and more, and that you agree to the terms as described.

If you have any questions or would like a copy of this consent letter, please contact:

Dr. FadwaHammouh,
Department of Nutrition and Dietetics,
Faculty of Health Sciences,
American University of Madaba, Jordan.

Email: f.hammouh@aum.edu.jo

Thank you in advance for your participation!

*Note: Research assistant will help you to understand and fill the questionnaire, and this needs 20 minutes from your time which we really appreciate.

Consent form of participation

[ ] I agree
[ ] I don't agree

Signature of participant: **Date:**

Basic Information Questionnaire

*This questionnaire aims to study the extent of Nutritional Knowledge, Attitudes and Practices of Elderly Jordanians.
* All information shall be filled out by the participants or a relative of them in the event of inability to write, under the supervision of researchers, knowing that all information received will be treated with strict confidentiality, and in the event of publication, the name will not be included in the search at all.

Serial Number: ------------------ Date: -------------------

1- Socio-Demographic Questions

* Governorate: -------------------------- * Place of Residence: ( ) Rural ( ) Urban
*Nationality: ------------------------- *Gender: ( ) Male ( ) Female
* Marital Status: ( ) Married ( ) Single ( ) Divorced ( ) Widowed
* Educational Status: ( ) Illiterate ( ) Tawjihi (high school) ( ) Diploma ( ) Bachelor ( ) More
* Residence: ( ) Alone ( ) With Spouse/Family
* Family No: ( ) Males ( ) Females
* Age (years): ( ) 60-69 yrs ( ) 70-79 yrs ( ) 80 yrs and above
* Weight (Kg)= ---------------- *Last time you weighed yourself: ------------------
*Where you weighed yourself: ---------------
* Height (m)= ----------------- *Last time you measured your height: ---------------
*Where you measured your height: -----------------
*Do you work? ( ) Yes ( ) No *If yes, specify? --
* Monthly family income (JD): ( ) Less than 500 ( ) 500- 1000 ( ) More than 1000
*Source(s) of income: ( ) work ( ) Sons/daughters ( ) Retirement salary (specify)
( ) National Aid Fund (specify?)
* Do you smoke cigarettes? ( ) Yes ( ) No
* If yes, number of cigarettes you smoke daily? ----------------------
* Do you smoke Hookah? ( ) Yes ( ) No
* If yes, number of weekly hookah smoking? -----------------------
* Is it allowed for elderly to practice exercises like walking, swimming and weight lifting? ( ) Yes ( ) No

*Meals you eat daily? Specify in the following table:

Meal	Yes	No	With Whom you eat it?
Breakfast			
Snack			
Lunch			
Snack			
Dinner			
Snack			

2-Health Information

*Do you suffer from tooth problems? ( ) Yes ( ) No
If yes, specify the problem: --
Do you have dentures or part of dentures? ( ) Yes ( ) No

*Do you have vision problems? ( ) Yes ( ) No

If yes, specify the problem: (   ) Long- sighted   (   ) Short- sighted   (   ) Glaucoma
(   ) Others, Specify: -------------------------------
*Do you suffer from chronic diseases? (   ) Yes   (   ) No

Third: Knowledge Questions

No	Question	YES	NO
1	Do you know malnutrition is a disease?		
2	Do you know malnutrition have complications?		
3	Do you know symptoms of malnutrition?		
4	Do you know the body mass index (BMI)?		
5	Do you know the normal level of body mass index?		
6	Is the junk food harmful to men?		
7	Is the junk food harmful to women?		
8	Is obesity considered malnutrition?		
9	Are there benefits for exercise in malnutrition?		

Fourth: Attitude Questions

No	Question	Strictly Agree	Agree	Neutral	Disagree	Strictly Disagree
1	Calculating BMI is essential					
2	It is mandatory to visit a physician periodically without being sick					
3	I should weigh myself periodically					
4	I should evaluate food quality in terms of quality and quantity					
5	Nutritional health is as important as mental health					
6	Good nutrition is essential in diseases as medicine					
7	Dinner is as essential as breakfast					
8	I have to eat breakfast every day					
9	Doing weight-lifting, warm- up and stretching exercises are essential daily					
10	It is essential to eat olive oil daily					
11	It is essential to eat more than 3 servings of vegetables daily					
12	It is essential to drink at least 4 cups of water					
13	Ghee and animal fats should be eaten with cautiously and sparingly					
14	I think that sugars and oriental sweets are empty foods of proteins, vitamins and minerals					
15	I think that honey and dates should not be used in excess					
16	I trust that bran bread and grain bread are better than white bread					
17	Read the leaflets that come with the medicine to avoid complications					

18	I know that drug complications are a cause malnutrition or a reason for suppressing appetite					
19	I understand that calcium is essential for bones, as magnesium, vitamin D and iron storage					

Fifth: Practices Questions

No	Question	YES	NO
1	Should you reduce fats and fast food in your meals?		
2	Do you check your BMI regularly?		
3	Do you visit a doctor regularly even if you do not suffer from disease or any complains?		
4	Do you exercise regularly 30 minutes/5 days a week or more?		
5	Are you keen to eat breakfast daily?		
6	Do you eat 3 main meals?		
7	Are you interested in taking supplements (vitamins or minerals)?		
8	Do you read the food label to know the content of calories, fats, sugars and other nutrients?		
9	Do you adhere to medication appointments and consider compliance in the event of interference with food?		
