# Supplementary material for: Nutrition Knowledge, Attitudes, and Practices (KAPs) among Jordanian Elderly—A Cross-Sectional Study
Source: Nutrients. 2023 May 8;15(9):2220. doi: 10.3390/nu15092220 (PMC10181286; doi:10.3390/nu15092220)
Supplement: Supplementary file 1 [file nutrients-15-02220-s001.zip › Supplementary File S2.pdf]

<u>Supplementary File- S2</u>

<u>الجامعة الأميركية في مادبا</u>

<u>كلية العلوم الصحية/ قسم التغذية والحميات</u>

<u>تقييم مدى المعرفة والاتجاه الإدراكي والممارسات التغذوية لدى كبار السن من الأردنيين/ دراسة مستعرضة</u>

موافقة لجنة المراجعة المؤسسية رقم: ----------

نموذج الموافقةعلى المشاركة

السادة /السيدات المشاركين في الاستبانة المحترمين
تعد المشاركة في هذه الدراسة اختيارية تمامًا. وإذا قررت عدم المشاركة فلن تكون هناك أي نتائج سلبية.
سيحافظ الباحث على سرية المعلومات، وجميع البيانات ستكون مجهولة دون أي إشارة إلى هوية المشارك.
من خلال تقديم هذا النموذج، فهذا يشير إلى أنك قد قرأت وصف الدراسة، وأنك في عمر 60 عاما وأكبر،
وأنك توافق على الشروط الواردة فيها كما هو موض□.

إذا كانت لديك أي أسئلة أو ترغب في الحصول على نسخة من خطاب الموافقة هذا،فيرجى الاتصال
مع الدكتورة فدوى حموه، قسم التغذية والحميات، كلية العلوم الصحية، الجامعة الأمريكية في مادبا، الأردن
البريدالإلكتروني: f.hammouh@aum.edu.jo

شكرا لكم مقدما على مشاركتكم!

*ملاحظة: سيقوم مساعد الباحث□مساعدتك في مليء الاستبانة وهذا يستغرق 20□قيقة من وقتك وهذا محل تقديرنا.

<u>*نموذج الموافقةعلى المشاركة</u>

(   ) أوافق

(   ) لا أوافق

توقيع المشار□:   التاريخ:

Basic Information Questionnaire
معلومات شخصية واجتماعية

*يهدف هذا الاستبيان إلى **تقييم مدى المعرفة والاتجاه الإدراكي والممارسات التغذوية لدى كبار السن من الأردنيين/ دراسة مستعرضة.**

*تعبأ جميع المعلومات من قبل المشتر□/المشتركة أو قريب لهما في حالة عدم القدرة على الكتا□ة وذلك□إشراف الباحثين، مع العلم أن كافة المعلومات الوار□ة ستعامل□سرية تامة، وفي حالة النشر لن ير□ الاسم في البحث إطلاقا

الرقم المتسلسل: --------------------   التاريخ: --------------------

<u>أولا: المعلومات الشخصية والديموغرافية:</u>

*المحافظة: ------------   *مكان السكن: ( ) قرية   (   ) مدينة

*الجنسية: ----------------   *الجنس: (   ) ذكر   (   ) أنثى

*الحالة الاجتماعية: ( ) متزوج   (   ) أعزب   (   ) مطلق   (   ) أرمل

*التعليم: (   ) غير متعلم   (   ) توجيهي   (   )□□لوم متوسط   (   )□كالوريوس   (   ) أعلى من ذلك

*السكن: (   ) لوحده/لوحدها   (   ) مع الزوج-الزوجة/ العائلة

*عد□ أفرا□ الأسرة: عد□ الذكور (   )   عد□ الإنا□ (   )

*العمر: (   ) 60-69 عاما   (   ) 70-79 عاما   (   ) 80 عاما وأكثر

*الوزن (كغم)= ----------------- *آخر مرة قمت□ها□توزين نفسك: ------------------

*أين قمت□توزين نفسك؟ --------------------

*الطول (متر)= ----------------*آخر مرة قمت□ها□قياس طولك: ------------------

*أين قمت□قياس طولك؟ --------------------

*هل تعمل؟ (   ) نعم   (   )لا ،إذا كانت الإجا□ة نعم، فحد□ عملك: ---

*الدخل الشهري للأسرة (□ينار أر□ني):

(   ) أقل من 500□ينار   (   ) 500- 1000□ينار   (   ) أكثر من 1000□ينار

*مصدر الدخل: (   ) العمل   (   ) الأولا□   (   ) راتب تقاعد (حد□ه)   (   ) معونة وطنية (حد□ مصدرها)

*هل تدخن السجائر؟ (   ) نعم   (   )لا، إذا كانت الإجا□ة نعم، عد□ السجائر التي تدخنها يوميا: ----------

*هل تدخن الأرجيلة؟ (   ) نعم   (   )لا، إذا كانت الإجا□ة نعم، عد□ مرات تدخين الأرجيلة أسبوعيا:------

*هل تشرب الكحول؟ (   ) نعم   (   )لا، إذا كانت الإجا□ة نعم، حد□ الكمية: ----------------------------

* هل يجوز لكبار السن ممارسة الرياضة مثل المشي والسباحة (   ) نعم   (   )لا

* الوجبات التي تتناولها يوميا؟ حد□ حسب الجدول التالي:

الوجبة	نعم	لا	مع من تناولت الوجبة؟
الإفطار			
وجبة خفيفة			
الغذاء			
وجبة خفيفة			
العشاء			
وجبة خفيفة			

<u>ثانيا: معلومات صحية</u>

*هل تعاني من مشاكل أسنان؟ (   ) نعم   (   ) لا

إذا كانت الإجا□ة نعم، حد□ المشكلة: --

*هل لديك الأسنان البديلة (الطارة كاملة أو جزء منها)؟ (   ) نعم   (   ) لا

*هل تعاني مشاكل في النظر؟ (   ) نعم   (   ) لا

إذا كانت الإجا□ة نعم، حد□ المشكلة: (   ) طول نظر   (   ) قصر نظر   (   ) الماء الزرقاء

(   ) غيرها ، حد□:-----------------------------

*هل تعاني من أمراض مزمنة؟ (   ) نعم   (   ) لا

ثالثا: أسئلة المعرفة

الرقم	السؤال	نعم	لا
1	هل تعلم أن سوء التغذية مرضا؟		
2	هل تعلم أن لسوء التغذية مضاعفات؟		
3	هل تعلم ما هي أعراض سوء التغذية؟		
4	هل تعلم ما هو مؤشر كتلة الجسم؟		
5	هل تعلم ما هو المستوى الطبيعي لمؤشر كتلة الجسم؟		
6	هل تعتبر الوجبات السريعة مؤذية للذكور؟		
7	هل تعتبر الوجبات السريعة مؤذية للإنا□ ؟		
8	هل تعتبر السمنة سوء تغذية؟		
9	هل للرياضة فائدة في حالة سوء التغذية؟		

رابعا: أسئلة الاتجاه الإدراكي

الرقم	السؤال	أوافق بشدة	أوافق	محايد	لا أوافق	لا أوافق بشدة
1	يعتبر حساب مؤشر كتلة الجسم مهم					
2	تعد زيارة الطبيب□صورة□ورية□دون ان اكون مريضا شيئا الزاميا					
3	انا اقتنع انني يجب ان اقوم□توزين نفسي□وريا					
4	انا اعتقد انني يجب ان اقوم□تقييم جو□ة غذائي من ناحية الكمية والنوعية					
5	الصحة التغذوية تساوي في الاهمية مع الصحة النفسية					
6	التغذية الجيدة مهمة في حالات المرض مثل الدواء					
7	وجبة العشاء مهمة مثل وجبة الفطور					
8	يجب ان اتناول الفطور يوميا					
9	يعتبر القيام□تمارين رفع الاثقال والاحماء والاطالة اساسية يوميا					
10	مهم ان اكل زيت الزيتون يوميا					
11	مهم ان اكل الخضروات اكثر من 3 حصص يوميا					
12	مهم ان اشرب على الاقل 4 كأس من الماء					
13	اعتقد ان اللية والسمن البلدي يجب تناولهما□حذر و□شكل ضئيل					
14	اعتقد ان السكريات والحلويات الشرقية اغذية فارغة من البروتينات والفيتامينات والمعا□ن					
15	اعتقد ان العسل والتمر لا يجب الاكثار منهما					
16	اثق ان خبز النخالة والخبز□الحبوب افضل من الخبز الا□يض					
17	يجب قراءة النشرات المرفقة مع الدواء حتى لا يحد□ مضاعفات					
18	اعرف ان المضاعفات الدوائية سبب في سوء التغذية او سبب في منع الشهية					
19	افهم ان الكالسيوم مهم للعظام و□اهمية المغنسيوم وفيتامين□ال ومخزون الحديد					

خامسا: اسئلة الممارسات:

الرقم	السؤال	نعم	لا

1	هل يجب تقليل الدهون والوجبات السريعة في طعامك؟		
2	هل تقوم□الفحص أو الحساب المنتظم لمؤشر كتلة الجسم؟		
3	هل تقوم□مراجعة الطبيب□انتظام حتى ولو لم تكن تعاني من مرض أو شكوى؟		
4	هل تمارس الرياضة□انتظام□واقع 30 □قيقة/ 5 أيام□الأسبوع أو أكثر؟		
5	هل تحرص على تناول الفطور يوميا؟		
6	هل تتناول 3 وجبات رئيسية؟		
7	هل تهتم□الحصول على مكملات غذائية (فيتامينات أو معا□ن)؟		
8	هل تقرأ□طاقة البيان للأغذية□حيث تعرف محتوى السعرات والدهون والسكر وغيرها؟		
9	هل تتقيد□مواعيد الدواء وتراعي الالتزام في حال وجو□ تداخل مع الغذاء؟		
